# Clinical Outcomes and Complications Following Subtotal Cholecystectomy: A Single-Centre Cohort Analysis

**DOI:** 10.7759/cureus.98477

**Published:** 2025-12-04

**Authors:** Oday Al-Asadi, Joussi Ramzi, Mostafa Mahran, Farah Aldhaher, Karim Ataya, Roham Karimi, Mohammad Yousef

**Affiliations:** 1 General Surgery, Homerton University Hospital, London, GBR; 2 General Surgery, North Middlesex University Hospital, London, GBR; 3 General and Upper Gastrointestinal (GI) Surgery, Ain Shams University, Cairo, EGY; 4 Upper Gastrointestinal (GI) Surgery, Homerton University Hospital, London, GBR; 5 Radiology, Guy's and St Thomas' NHS Foundation Trust, London, GBR; 6 Surgery, Homerton University Hospital, London, GBR

**Keywords:** bile leak, cholecystectomy outcomes, difficult gallbladder, laparoscopic surgery, subtotal cholecystectomy

## Abstract

Background

Subtotal cholecystectomy (STC) is increasingly used as a safe technique for managing difficult gallbladders to reduce the risk of common bile duct (CBD) injury. However, techniques and outcomes vary across institutions, and standardised reporting remains limited.

Objective

To evaluate peri-operative and post-operative outcomes following STC at a single tertiary centre and to explore pre-operative factors associated with the need for STC, including the frequency of previous gallstone-related admissions and radiologic markers such as impacted infundibular or neck stones, thickened or contracted gallbladder, and imaging features suggestive of a frozen Calot’s triangle.

Methods

Retrospective cohort study of consecutive patients undergoing STC between January 2021 and January 2024. Data included demographics, indications, operative technique (fenestrating vs. reconstituting), intra-operative findings, complications, and long-term biliary outcomes. The primary endpoint was clinically significant bile leak; secondary outcomes were reoperation, endoscopic retrograde cholangiopancreatography (ERCP) requirement, readmission, and mortality.

Results

Thirty-nine patients underwent STC (97% laparoscopic, 3% open). Median age 49 years with a mean BMI of 39 kg/m^2^. Bile leak occurred in 2.6% (n = 1), reoperation 0%, ERCP 2.6% (n = 1), readmission 0%, and mortality 0%.

Conclusions

STC offers a safe alternative to total cholecystectomy in complex cases, though bile leak remains an important morbidity. Technique type and intra-operative factors influence outcomes, highlighting the need for standardised approaches and careful case selection.

## Introduction

Background

Cholecystectomy is the definitive management for gallstone disease. In cases of severe inflammation or obscured anatomy at Calot’s triangle, STC is employed to prevent bile duct injury [1,2]. More generally, the term difficult gallbladder describes a cholecystectomy which is expected to be more difficult than a routine cholecystectomy. Although the incidence of difficult gallbladders varies among series, it may be estimated that one in six gallbladders is “difficult” [3].

Objectives

To describe the outcomes of STC at our centre, analyse results by technique, and identify predictors of the need for STC.

## Materials and methods

Study design and setting

This is a retrospective cohort analytic study conducted at Homerton University Hospital, our tertiary referral centre in London, United Kingdom. The study adhered to the Strengthening the Reporting of Observational Studies in Epidemiology (STROBE) guidelines [4].

All patients who underwent STC between January 2021 and January 2024 were included. Cases from emergency or elective settings were all included. All cases are performed by experienced upper gastrointestinal or emergency surgeons. A second opinion from another consultant colleague was taken when needed.

Patients <18 years, those undergoing total cholecystectomy, cases performed for malignancy, or those where cholecystectomy formed part of another operation were excluded.

Cases were identified from electronic theatre logs. Data were extracted from the hospital’s electronic patient records and operative notes. Demographic characteristics of patients, including age, sex, body mass index (BMI), and co-morbidities (e.g., diabetes, pancreatitis) were obtained.

Pre-operative data, including the number of previous admissions, were collected. The radiological findings (e.g., gallstones, wall thickness, impacted stones, ductal dilatation, Mirizzi syndrome, perforation) were analysed in detail and reviewed by a consultant radiologist. Detailed information on the intra-operative findings, e.g., frozen Calot’s triangle anatomy, degree of inflammation, adhesions, and conversion to open surgery, was checked to address the reasons for the intra-operative decision to perform a subtotal cholecystectomy as a bailout procedure rather than the planned laparoscopic cholecystectomy.

Operative details regarding the type of subtotal cholecystectomy have been investigated. We followed the Strasberg classification for subtotal cholecystectomy, which divides the procedure into two categories, fenestrating or reconstituting, depending on whether the gallbladder remnant is closed or left open [1]. The stump management technique used in our cases (e.g., suture closure, stapled closure, use of endo-loop, hemo-lock, or open stump), drain placement, as well as intra-operative complications were all analysed.

Post-operative outcomes, including 30-day morbidity and mortality, re-operation, post-operative endoscopic retrograde cholangiopancreatography/magnetic resonance cholangiopancreatography (ERCP/MRCP), rate of readmission, and length of stay, were also reviewed in detail. Persistent bilious drainage or imaging evidence requiring intervention is considered a clinically significant bile leak.

Follow-up was conducted between two to four years post-operatively via outpatient visits, virtual clinic notes, and review of hospital records for any gallbladder-related readmissions, interventions, or imaging.

Statistical analysis

Data were analysed using Microsoft Excel 2024 (Microsoft Corp., Redmond, WA, USA). Continuous variables are presented as mean ± standard deviation (SD) or median (interquartile range, IQR). Categorical variables are expressed as number (n) and percentage (%).

Ethical considerations

This study was approved by the hospital research and innovation team. All data were anonymised prior to analysis. No external funding was received, and the authors declare no conflicts of interest.

## Results

Cohort overview

Between January 2021 and January 2014, 778 cholecystectomies were performed, of which 39 (5.0%) were subtotal. Median patient age was 49 years (SD 13.7), 21 (53.8%) were female subjects. Mean BMI was 39.0 ± 7.5 kg/m^2^ (Table 1).

**Table 1:** Baseline demographics and clinical characteristics (n = 3)

Variable	Mean ± SD/n (%)	Range/details
Age (years)	49 ± 13.7	22 – 77
Sex (male/female)	18 (46.2%)/21 (53.8%)	—
BMI (kg/m^2^)	39.0 ± 7.5	26 – 55
Diabetes mellitus	3 (7.7%)	—
Previous pancreatitis	2 (5.1%)	—
≥ 1 previous admission	16 (41.0%)	0 – 5 admissions

Pre-, intra-, and post-operative findings

Many patients who underwent subtotal cholecystectomy had a history of repeated gallstone-related admissions, with a mean of 1.2 admissions (median 1, range 0-5). Overall, 41% of patients had experienced more than one admission prior to their operation. Three patients were diabetic, and two patients had previous pancreatitis.

Pre-operative radiological findings are shown in Table 2. These included gallstones (100%), thick-walled gallbladder (82%), impacted stones at gallbladder neck (20.5%), dilated bile ducts (10.2%), Mirizzi syndrome (7.6%), adenomyomatosis (7.6%), and perforation (2.5%). These radiologic features often correlated with the intra-operative finding of dense adhesions and frozen Calot’s anatomy.

**Table 2:** Pre-operative radiological findings (n = 39)

Imaging finding	n (%)
Gallstones	39 (100%)
Thickened gallbladder wall	32 (82.1%)
Impacted neck stone	8 (20.5%)
Dilated bile ducts	4 (10.3%)
Mirizzi syndrome	3 (7.7%)
Adenomyomatosis	3 (7.7%)
Gallbladder perforation	1 (2.6%)

The main indications for subtotal cholecystectomy included intra-operative findings of necrotic, emphysematous, perforated, frozen, or contracted gallbladders in which safe dissection of Calot’s triangle was not possible.

Several stump-management techniques were utilised depending on intra-operative findings and the surgeon’s judgement. Laparoscopic suturing was performed in 15 (38.4%) cases, endo-loop ligation in 11 (28.2%), stapler closure was used in 10 (25.6%), large hemo-lock in two (5.1%) and the stump was left open in one case (2.6%). Drains were placed in 33 patients (85%), with six patients left without drainage at the surgeon’s discretion.

Conversion to open surgery occurred in one case (2.6%) due to bleeding from the staple line. This was a tan-coloured tri-stapler reload. At laparotomy, bleeding from the gallbladder stump edge was controlled successfully without further complications. The details are illustrated in Table 3.

**Table 3:** Intra-operative findings and stump management techniques (n = 39) STC: subtotal cholecystectomy

Parameter	n (%)	Notes
Frozen Calot’s/dense adhesions	28 (71.8%)	—
Necrotic/perforated gallbladder	5 (12.8%)	—
Contracted gallbladder	6 (15.4%)	—
Laparoscopic suturing	15 (38.4%)	Reconstituting STC
Endoloop ligation	11 (28.2%)	Reconstituting STC
Stapler closure	10 (25.6%)	Reconstituting STC
Hemo-lock clip	2 (5.1%)	Reconstituting STC
Open stump (fenestrating)	1 (2.6%)	—
Drain placement	33 (84.6%)	—
Conversion to open surgery	1 (2.6%)	Staple-line bleeding

The mean length of hospital stay was 3.6 ± 3.6 days. Post-operative complications were infrequent and included a single bile leak (2.6%) and three intra-abdominal collections (7.7%), all managed conservatively or with image-guided drainage. Post-operative imaging and endoscopic interventions were required in a small proportion of patients, with ERCP performed in 2.6% for post-operative bile leak and MRCP in 7.7% (Table 4).

**Table 4:** Post-operative outcomes and complications (n = 39)

Outcome	n (%)	Management
Bile leak	1 (2.6%)	Managed conservatively with drain and ERCP
Intra-abdominal collection	3 (7.7%)	Image-guided drainage
ERCP performed	1 (2.6%)	For post-operative bile leak
MRCP performed	3 (7.7%)	For follow-up imaging
Conversion to open surgery	1 (2.6%)	Controlled bleeding
Readmission	0 (0%)	—
Reoperation	0 (0%)	—
Mortality	0 (0%)	—
Mean length of stay (days)	3.6 ± 3.6	Range 1 – 19

Discharge criteria included clinical stability, normal liver function test and minimal drain output. Histopathological analysis predominantly demonstrated chronic cholecystitis in 30 patients (approximately 77%), with occasional acute-on-chronic inflammation or mild dysplastic changes. During follow-up, a small number of patients experienced transient abdominal pain or small remnant collections; all resolved spontaneously or normalised on subsequent imaging.

There were no bile duct injuries, readmissions, bile duct injuries or mortality cases in our series. Details of histopathological results are demonstrated in Table 5.

**Table 5:** Histopathological findings (n = 39)

Histopathology result	n (%)
Chronic cholecystitis	30 (76.9%)
Acute-on-chronic inflammation	7 (17.9%)
Mild dysplastic changes	2 (5.1%)
Malignancy	0 (0%)

## Discussion

Laparoscopic subtotal cholecystectomy (STC) seems to be seen frequently in current surgical practice, with an average of one case per month in our series of 39 patients in three years. There was no special correlation with pre-operative history of diabetes or pancreatitis. However, our study suggested that the majority of the patients were at the high BMI side, classified as class 2 obesity (Mean BMI is 39 kg/m^2^).

Principal findings

Conversion to open surgery was required in only 2.6% of cases. Morbidity was low, with a bile leak rate of 2.6% and intra-abdominal collections in 7.7%. These outcomes compare favourably with published series [5-7]. The patient who needed conversion to open surgery had his gallbladder stump managed with a tan-coloured linear stapler. He bled more than 1 litre of blood, where conversion to open surgery was needed to get a better view and put an overstitch on the stapler edge where the bleeding point was. The single patient who developed post-operative bile leak had his gallbladder stump managed with a laparoscopic suturing technique. The bile leak was controlled by the presence of the sub-hepatic drain. The patient was treated with post-operative ERCP successfully. However, he remained in the hospital for a longer period (19 days). The mean length of stay for the cohort was 3.6 days; excluding the single patient with a prolonged 19-day admission due to bile leak, the mean was 3.1 days. Both values exceed the usual same-day discharge associated with routine laparoscopic cholecystectomy. Histology confirmed predominantly chronic inflammation, consistent with “frozen” Calot’s anatomy [1,6].

Comparison with the literature

Systematic reviews report bile leak rates between 10% and 20% and conversion rates of around 7% [5-8]. Fenestrating techniques carry a higher leak risk (odds ratio ≈ 2.5) than reconstituting techniques [6,9]. Our low bile leak rate may reflect careful intra-operative decision-making, secure stump closure, and tailored gallbladder stump management techniques to individual cases.

Multicentre studies and meta-analyses [9-11] confirm similar trends (Table 6).

**Table 6:** Summary of outcomes compared to literature

Study	Year	No. of cases	Bile leak (%)	Conversion to open (%)
Present study	2025	39	2.6	2.6
Strasberg et al. [1]	2016	34	8.0	5.0
Al-Azzawi et al. [5]	2024	212	12.0	6.8
Ravendran et al. [7]	2024	681	10.0–20.0	7.0
van Dijk et al. [12]	2017	54	11.0	9.0

Strengths and limitations

Strengths include real-world single-centre data reflecting NHS practice, a clearly defined classification of stump management techniques, and near-complete follow-up. Limitations include retrospective design, lack of standardised long-term imaging, and limited generalisability beyond a single institution.

Clinical implications

Frequent readmissions, high BMI and imaging findings such as an impacted infundibular stone, a thick-walled or contracted gallbladder, should alert surgeons to the likelihood of requiring STC. Awareness of these predictors can improve pre-operative counselling, intra-operative planning, and post-operative bed management. Structured follow-up, including liver-function monitoring, drain assessment, and early ultrasound or MRCP, when indicated, facilitates timely detection of bile leaks [9,11] **[10,12]**.

Future directions

Future studies should focus on prospective multicentre registries to standardise definitions, techniques, and long-term outcome reporting [6,12].

## Conclusions

Subtotal cholecystectomy remains a safe and reliable bailout procedure for difficult gallbladders, associated with low rates of bile leak and conversion to open surgery. Our study reported zero rate of readmission, reoperation, bile duct injury or mortality. Careful technique selection, secure stump closure whenever possible, and structured post-operative monitoring are key to optimising outcomes.
